# EARLY-LIFE INVOLUNTARY JOB LOSS AND LATER-LIFE DEPRESSIVE SYMPTOMS IN CHINA: A TRAJECTORY ANALYSIS

**DOI:** 10.1093/geroni/igae098.2672

**Published:** 2024-12-31

**Authors:** Qian Song, Wuyi Dong

**Affiliations:** University of Massachusetts Boston, Boston, Massachusetts, United States; University of Massachusetts Boston, Boston, Massachusetts, United States

## Abstract

While it is relatively well-understood that involuntary job loss (job loss thereafter) has short-term negative impacts on health of individuals, we do not know much about how early-life job loss is associated with long-term depressive symptoms, especially in the developing country contexts. In this study, we take advantage of a nationally representative sample for those aged 45 and older in the 2011-2018 China Health and Retirement Longitudinal Study as well as the Life History data collected in 2014. We investigate how early life job loss experience, much of which occurred more than two decades ago, is associated with depressive symptoms, as measured by CESD-10 (range: 0-30), in mid- and later-life. Among our sample (N=3,653), 32% of Chinese have experienced job loss at least once in their life-time. After controlling for gender, age, education, marital status, household expenditure, self-rated health and activities of daily living, we found that those who have experienced job loss showed an increased (β=.53, p<.001) number of depressive symptoms at the age of 60, and with one year of increase in age, they experienced an additional.02 (p<.05) growth compared to those who never experienced job loss. The timing of job loss matters. Job loss occurred during mid-career (30-45 years old) was most detrimental to later-life psychological well-being, with.98 (p<.001) more depressive symptoms at age 60 and an additional.06 growth (p<.05) with each year. The results highlight the long arm of early-life employment experience on the depressive symptoms of individuals even decades after.

